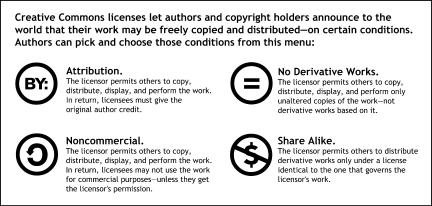# Out of the Way: How the next copyright revolution can help the next scientific revolution

**DOI:** 10.1371/journal.pbio.0000009

**Published:** 2003-10-13

**Authors:** Glenn Otis Brown

## Abstract

By default, all published works are copyrighted. Creative Commons provides means for authors to share their work more freely

When did calling a lawyer become part of the scientific process? It hasn't officially, of course. But as a generation of researchers has grudgingly come to know, navigating the legal red tape of universities, corporations, and publishers is an inevitable part of the practice. Whether seeking access to information or sharing one's own findings with others, scientists increasingly find themselves having to ask an intermediary's permission.

This “ask first” culture has developed at just the moment when technology has opened vast new possibilities for collaboration and information-sharing. The timing is not coincidental. Policymakers, under the influence of lobbies defending pre-digital business models, have reacted to new technology with ever more extreme intellectual property laws. The result is a legal regime tailored to a powerful minority but ill-suited to a number of other constituencies—scientists and scholars chief among them—that thrive on openness. Worries over broad notions of “piracy” and “asset management” have insinuated themselves into fields where those terms, until recently, held no meaning.

The Public Library of Science (PLoS) is at the vanguard of a growing cross-disciplinary movement to counteract this trend by demonstrating that voluntary models of open publishing are not only viable, but crucial to scientific innovation. Yet PLoS’ goal of “immediate, unrestricted access to scientific ideas, methods, results, and conclusions” is not immediately compatible with the stringent rules of copyright, which apply fully and automatically to all published works, by default. The exercise of something less than full copyright requires, oddly, some legal tinkering—which is where Creative Commons, the organization I help manage, comes in.

Creative Commons, a 501 (c) (3) nonprofit corporation based at Stanford University in California, is led by a board of expert legal and technical thinkers. (Its chairman, Lawrence Lessig, a law professor at Stanford and a recipient of the *Scientific American* 50 Award in 2003, recently joined the PLoS board of directors.) Creative Commons was founded on the idea that some people prefer to share their works on more generous terms than standard copyright provides. The organization offers such authors an easy and clear way to announce these preferences. The goal is to help endeavors like PLoS, as well as individual authors, expand access to quality content online while reducing the legal friction and uncertainty of copyright law. In other words, Creative Commons offers legal tools to help clear permissions, once and for all. We help get the law out of science's way.

These tools are the Creative Commons licenses, a suite of form legal documents available for free on the Creative Commons website. Each license allows an author to retain his or her copyright while permitting certain uses of the work, on certain conditions: to declare “some rights reserved” as opposed to “all rights reserved.” From a simple menu, copyright holders mix and match their preferences: an attribution requirement; a prohibition on commercial reuse; a restriction on derivative works; or a “share-alike” provision that obligates licensees to offer any derivative works to the public on the terms they received. (PLoS has chosen the simplest and least restrictive of the licenses, permitting copying, as well as free commercial reuse and transformation, in exchange for simple attribution.)

Since the licenses’ launch in December 2002, nearly 800,000 Web pages (well over 1,000,000 discrete works) have been made available under Creative Commons licenses. Because they're free and can apply to any kind of copyrighted work, the licenses have been popular with Webloggers, teachers, novelists, musicians, photographers, and hobbyists. Many institutional adopters, too, have used the licenses to facilitate innovative publishing techniques, particularly in the sciences.

The Massachusetts Institute of Technology's Open Courseware project publishes materials from its university courses under a version of the licenses, inviting students and educators from around the world to reuse them royalty-free. Rice University's Connexions project, an interactive tool that helps instructors build courses and texts from a collective knowledge repository, requires authors to license their contributions for free reuse in return for authorial attribution. The American Museum of Natural History's Biodiversity Commons will soon use the licenses to facilitate search across a broad collection of conservation databases and websites.

Like PLoS, all of these projects use Creative Commons licenses to simplify and streamline the process of rights clearance. But the licenses also serve another critical function: they formalize the collaborative ethos of the scientific and academic communities in a language that legal intermediaries cannot quarrel with. This standardization also helps otherwise disparate communities, whether across disciplines or geographic boundaries, to agree in advance on the rules for sharing.

Creative Commons is now considering expanding into other fields where the law has begun to restrict open research: scientific data and patents, in particular. With a portion of a new US$1 million grant from the Hewlett Foundation (putting our total of funding received at over US$3 million), we hope to build the Science Commons, a branch of the organization dedicated to bringing a measure of reason, and restraint, to the legal thicket that has grown around scientific research.

Like PLoS, Creative Commons’ goals and methods are designed to make the most of the opportunities created by new communications technology. But, also like PLoS, our inspiration reflects the wisdom and optimism of the Enlightenment as much as that of the Digital Age. We are trying to restore the sense of legal moderation that policymakers of a bygone era, heavily influenced by the philosophy of the first scientific revolution, understood would “promote the progress of science and the useful arts,” as the U.S. Constitution puts it.

“Knowledge [is] not the personal property of its discoverer, but the common property of all,” wrote Benjamin Franklin, the great cosmopolitan, polymath, and patron saint of innovation. As we enjoy great advantages from the inventions of others, we should be glad of an opportunity to serve others by any invention of ours, and this we should do freely and generously.”

Franklin, who knew a thing or two about both publishing and science, never practiced law.

**Figure:**